# Supplementary material for: Theta mediated dynamics of human hippocampal-neocortical learning systems in memory formation and retrieval
Source: Nat Commun. 2023 Dec 21;14:8505. doi: 10.1038/s41467-023-44011-6 (PMC10739909; doi:10.1038/s41467-023-44011-6)
Supplement: Supplementary file 2 — Reporting Summary [file 41467_2023_44011_MOESM2_ESM.pdf]

nature portfolio

Corresponding author(s): Michael Yassa

Last updated by author(s): Oct 12, 2023

Reporting Summary

Nature Portfolio wishes to improve the reproducibility of the work that we publish. This form provides structure for consistency and transparency in reporting. For further information on Nature Portfolio policies, see our Editorial Policies and the Editorial Policy Checklist.

Statistics

For all statistical analyses, confirm that the following items are present in the figure legend, table legend, main text, or Methods section.

n/a	Confirmed	
☐	☒	The exact sample size (n) for each experimental group/condition, given as a discrete number and unit of measurement
☐	☒	A statement on whether measurements were taken from distinct samples or whether the same sample was measured repeatedly
☐	☒	The statistical test(s) used AND whether they are one- or two-sided<br>*Only common tests should be described solely by name; describe more complex techniques in the Methods section.*
☐	☒	A description of all covariates tested
☐	☒	A description of any assumptions or corrections, such as tests of normality and adjustment for multiple comparisons
☐	☒	A full description of the statistical parameters including central tendency (e.g. means) or other basic estimates (e.g. regression coefficient) AND variation (e.g. standard deviation) or associated estimates of uncertainty (e.g. confidence intervals)
☐	☒	For null hypothesis testing, the test statistic (e.g. F, t, r) with confidence intervals, effect sizes, degrees of freedom and P value noted<br>*Give P values as exact values whenever suitable.*
☐	☒	For Bayesian analysis, information on the choice of priors and Markov chain Monte Carlo settings
☐	☒	For hierarchical and complex designs, identification of the appropriate level for tests and full reporting of outcomes
☒	☐	Estimates of effect sizes (e.g. Cohen's d, Pearson's r), indicating how they were calculated

Our web collection on statistics for biologists contains articles on many of the points above.

Software and code

Policy information about availability of computer code

Data collection	The task was programmed in PsychoPy2 (Version 1.82.01).
Data analysis	Following acquisition, data were pre-processed using customized MATLAB scripts. Overlaid MRI images were visualized in AFNI for electrode localization. The labeled in-house template was resampled (1mm isotropic) and aligned to each subject's pre-implantation scans using ANTs Symmetric Normalization. Contacts from each subject meeting each of the inclusion criteria were projected onto a standard brain model (Montreal Neurological Institute, MNI) and visualized using DSI Studio. For spectral analysis, Analytic Morlet Wavelets were generated in MATLAB using the Wavelet toolbox. All Code and Statistics files are on GitHub at https://github.com/Yassa-TNL/theta_physiology for open data sharing and to enhance reproducibility.

For manuscripts utilizing custom algorithms or software that are central to the research but not yet described in published literature, software must be made available to editors and reviewers. We strongly encourage code deposition in a community repository (e.g. GitHub). See the Nature Portfolio guidelines for submitting code & software for further information.

Data

Policy information about availability of data

All manuscripts must include a data availability statement. This statement should provide the following information, where applicable:

- Accession codes, unique identifiers, or web links for publicly available datasets
- A description of any restrictions on data availability
- For clinical datasets or third party data, please ensure that the statement adheres to our policy

All data from this manuscript is available on Open Science Framework (OSF) at the following repository address: https://osf.io/dn9as/.

Field-specific reporting

Please select the one below that is the best fit for your research. If you are not sure, read the appropriate sections before making your selection.

☒ Life sciences ☐ Behavioural & social sciences ☐ Ecological, evolutionary & environmental sciences

For a reference copy of the document with all sections, see nature.com/documents/nr-reporting-summary-flat.pdf

Life sciences study design

All studies must disclose on these points even when the disclosure is negative.

Sample size	Electrophysiological recordings were acquired from eight adult (ages 23-52) patients (3 females) stereotactically implanted with depth electrodes (Integra or Ad-Tech, 5-mm interelectrode spacing) for clinical monitoring (Table 1). This is a convenience sample limited by the availability of a specific subject pool.
Data exclusions	The inclusion criterion was concurrent electrode coverage over both hippocampal (HC) and neocortical (NC) areas.
Replication	Within participant analyses were conducted and analyses replicated across participants to ensure robustness and reproducibility. In most analyses, technical replicates were used which involved either pooled trials or pooled channels across subjects. We could not use biologically independent replicates (subjects) given the limitation of obtaining a sufficient sample size with intracranial electrophysiological recordings.
Randomization	None. Subject selection and coverage is clinically determined and limited by availability of patients in epilepsy monitoring unit.
Blinding	None. Subject selection and coverage is clinically determined and clinicians are on the study team.

Reporting for specific materials, systems and methods

We require information from authors about some types of materials, experimental systems and methods used in many studies. Here, indicate whether each material, system or method listed is relevant to your study. If you are not sure if a list item applies to your research, read the appropriate section before selecting a response.

Materials & experimental systems

n/a	Involved in the study	
☒	☐	Antibodies
☒	☐	Eukaryotic cell lines
☒	☐	Palaeontology and archaeology
☒	☐	Animals and other organisms
☐	☒	Human research participants
☒	☐	Clinical data
☒	☐	Dual use research of concern

Methods

n/a	Involved in the study	
☒	☐	ChIP-seq
☒	☐	Flow cytometry
☒	☐	MRI-based neuroimaging

Human research participants

Policy information about studies involving human research participants

Population characteristics	Electrophysiological recordings were acquired from eight adult (ages 23-52) patients (3 females) stereotactically implanted with depth electrodes (Integra or Ad-Tech, 5-mm interelectrode spacing) for clinical monitoring (Table 1).
Recruitment	Participants were patients in the epilepsy monitoring unit. A combination of the neurosurgeon and the neurologist team inform the patient about the availability of research studies during the monitoring period and, if the participant is interested, they are screened for the study. The team clearly indicates to the patient that non-participation in the study will not impact their clinical care. The inclusion criterion was concurrent electrode coverage over both hippocampal and neocortical areas.
Ethics oversight	Informed consent was obtained from each participant for study inclusion in accordance with the institutional review board of the University of California, Irvine

Note that full information on the approval of the study protocol must also be provided in the manuscript.
